# Allergenicity of Fermented Foods: Emphasis on Seeds Protein-Based Products

**DOI:** 10.3390/foods9060792

**Published:** 2020-06-16

**Authors:** Kamel-Eddine El Mecherfi, Svetoslav Dimitrov Todorov, Marcela Albuquerque Cavalcanti de Albuquerque, Sandra Denery-Papini, Roberta Lupi, Thomas Haertlé, Bernadette Dora Gombossy de Melo Franco, Colette Larré

**Affiliations:** 1 INRAE UR1268 BIA, 3 impasse Y. Cauchois—Rue de la Géraudière CS 71627, 44000 Nantes, France; kamel-eddine.el-mecherfi@inrae.fr (K.-E.E.M.); sandra.denery@inrae.fr (S.D.-P.); robertinalupi@hotmail.it (R.L.); tom@haertle.fr (T.H.); 2 Food Research Center, Department of Food and Experimental Nutrition, Faculty of Pharmaceutical Sciences, University of Sao Paulo, Sao Paulo 05508-080, Brazil; slavi310570@abv.bg (S.D.T.); malbuquerque17@gmail.com (M.A.C.d.A.); bfranco@usp.br (B.D.G.d.M.F.)

**Keywords:** food allergens, seed proteins, fermentation, lactic acid bacteria, food allergy

## Abstract

Food allergy is an IgE-mediated abnormal response to otherwise harmless food proteins, affecting between 5% and 10% of the world preschool children population and 1% to 5% adults. Several physical, chemical, and biotechnological approaches have been used to reduce the allergenicity of food allergens. Fermentation processes that contribute to technological and desirable changes in taste, flavor, digestibility, and texture of food products constitute one of these approaches. Lactic acid bacteria (LAB), used as starter cultures in dairy products, are a subject of increasing interest in fermentation of plant proteins. However, the studies designed to assess the impact of LAB on reduction of allergenicity of seed proteins are at an early stage. This review presents the current knowledge on food fermentation, with a focus on seed proteins that are increasingly used as ingredients, and its impacts on food potential allergenicity.

## 1. Introduction

Food allergy is a public health concern affecting between 2% and 5% of the general population (1–5% adults and 4–8% children). This physiological disorder degrades significantly life quality of allergic individuals and sometimes can provoke severe health problems [1,2,3]. Hence, it presents a considerable economic burden for families and societies [4].

Food allergy is an adverse reaction to otherwise harmless food proteins, termed allergens, that involves an abnormal defensive response of the immune system. Allergic reactions preferably occur in atopic subjects, and repeated exposures are necessary to induce clinical symptoms [5].

**Figure 1:**
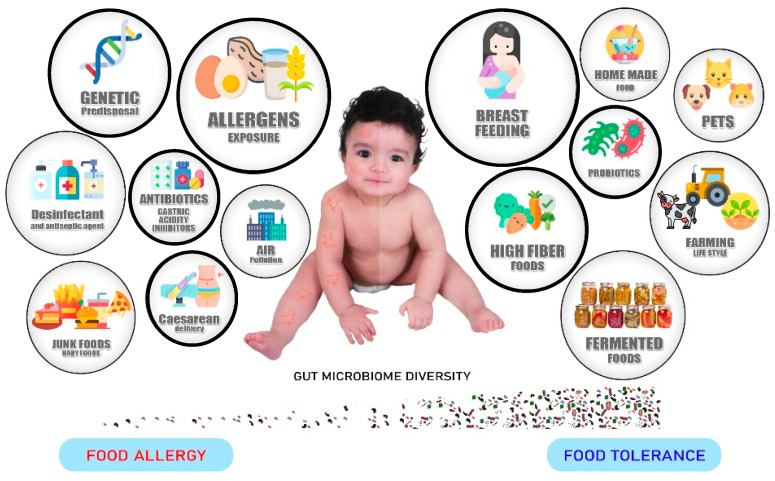
Known (bold ring) and suspected lifestyle factors (thin ring) affecting gut microbiome biodiversity. Factors mentioned on the left of the figure predispose to the development of food allergy and those on the right reinforce food tolerance [22].

While it is clear that this pathology is linked to atopic family history and to genetic predisposition, other nongenetic factors contribute significantly to its development and play an essential role in shaping the immune system and a loss of food tolerance. These factors are related mainly to the lifestyle in Western countries, including the nature and composition of the diet and exposure to pollution. Also, “the old friends hypothesis” proposes that loss of the commensal relationship with parasites and bacteria, in consequence of facts such as birth by cesarean instead of vaginal delivery, life in urban instead of rural environments, and abusive use of prescribed antibiotics as well as residual antibiotics in meat and fish food impact the microbial diversity in the human gut, disrupting the immunoregulatory function of the microbiota and predisposing to allergy (Figure 1) [6].

Nowadays, there is no practical method to abolish or eradicate food allergy reactions, and the most effective way to manage food allergy is to avoid contact or eliminate this food from the diet. An alternative to decrease the allergenicity of allergens in foods is processing, such as thermal treatment and fermentation. Over the centuries, new processes have emerged, moving from laboratory and semi-industrial scales to industrial stages, aiming at feeding a large number of people with more edible, healthy, and palatable products. The industrial revolution in the past century impacted not only society, but also changed the techniques of food production, and resulted in the emergence of a new type of processed food, reconstituted from isolated nutrients and added of a myriad of ingredients and additives. This category of new foods has raised many questions related to health and sustainability [7,8,9,10,11,12], particularly from an allergenicity point of view, as these foods may contain one or more allergens. It is now established that processing techniques, either old or new ones, could have positive or negative effects on the allergenic potential of a food, in consequence of modifications in the structure of allergens at molecular level (hydrolysis, aggregation, deamidation) and interactions with other food components (formation of complexes, Maillard reaction) [13,14,15,16,17,18].

In our modern societies, processed foods are being perceived as less healthy, leading consumers to turn away from them. However, fermentation is considered a natural process, and consumers have a positive perception of its impact on health [19,20]. Fermentation is used in the preparation of numerous food products, resulting in new organoleptic, gastronomic, nutritional, or functional properties. There are currently over 250 types of fermented products in the world, but the exact number is hard to estimate [21].

This review addresses the impact of fermentation on the allergenic properties of food proteins. As fermented dairy products are the most consumed, the review addresses the recent findings on the effect of this type of fermentation on milk proteins allergenicity, and then moves to seeds protein-based fermented foods, which are of increasing interest to consumers in parallel to progressing a nutrition transition to more sustainable diets and vegetarian style. The possibilities offered by the proteolytic system of lactic acid bacteria (LAB) to modify food proteins and, therefore, allergens, are discussed. In the final part, the review gives some examples of fermented products and their possible probiotic roles in modulation of the immune system and then of the allergic reactions.

## 2. Food Allergy

An allergic reaction consists of a hidden sensitization phase followed by a symptomatic phase called elicitation. Food allergy most often corresponds to a type I hypersensitivity (mediated by type E immunoglobulins, IgE) toward proteins of animal or plant origin (allergens). These allergens bind to IgE, on the surface of basophils or mast cells, and cause the release of pro-inflammatory mediators, such as histamine, and induce the symptomatic phase of allergy. The only symptoms’ treatment that has proven to be effective is the avoidance of the allergens, but this requires rigorous discipline in the control of the food intake. The risk is more substantial for consumers of processed products, which often contain a variety of ingredients, listed in labels that are not always easy to interpret [23].

### 2.1. Allergic Reactions and Prevalence

The epidemiology of food allergy has progressed considerably in recent years, producing an increasing amount of data on the prevalence of this disease, mostly in developed countries [3,24,25]. In contrast, there is little reliable data from developing countries where food allergies are very often misdiagnosed and even evaluated and treated negligently [26]. Additionally, the estimated prevalence of food allergy differs considerably depending on the applied survey methods. Population surveys based on self-assessment or parental reports produce results that vastly overestimate prevalence, at up to 20% [27]. A straightforward and reliable diagnosis of food allergy can be obtained by an oral challenge with clinical monitoring of exposed patients, but this procedure is costly and hard to implement in epidemiological surveys. Other methods, such as in vitro detection of specific IgE antibodies or skin prick tests (ST), are also used, even if less reliable. In skin prick tests, the quality of the response is determined by the structure and the quantity of the allergens used in the tests. For example, the use of fresh foods, compared with purified allergen extracts from milk, wheat, soy, egg, peanut, and kiwi, increases the effectiveness of the tests in predicting clinical reactivity [28].

In developed countries, an increasing trend in the prevalence of food allergies has been reported [3], reaching 8% in children and 5% in adults in 2014. The overall prevalence varies with the age of the patients and the type of allergen. Eight types of food are responsible for about 90% of food allergies: milk, egg, fish, shellfish, nuts, peanuts, wheat, and soy. Children are more often sensitized to milk and eggs, while adults respond more to plant-based foods such as peanuts or nuts. The estimated prevalence of each of these food allergies varies according to the cohort, country, rural or urban areas, and measurement methodology. Nevertheless, in the USA, the three types of foods responsible for the greatest number of allergies are shellfish (1.9%), fruits (1.6%), and vegetables (1.3%) [25]. Moreover, a recent analysis of the PEAR (Partners’ Enterprise-wide Allergy Repository) database in the USA confirmed that the most frequently recognized food allergies were caused by shellfish (0.9%), fruits and vegetables (0.7%), dairy products (0.5%), peanuts (0.5%), and nuts (0.4%) [29].

### 2.2. Seed Protein Allergens

The main plant proteins consumed to reduce animal protein intake come from seeds of cereals, legumes, and pseudo-cereals. Among these, wheat, lupine, mustard, sesame, soy, and their derivatives have been identified as allergenic food ingredients involved in severe or frequent reactions and they should be labeled as such. These allergens belong to a small number of protein families reported in Table 1, which gives an overview of the allergens identified in fifteen types of seeds. Table 1 indicates that 2S storage proteins, when present in seeds, are always allergenic, and that profilin-type allergens have been identified in all seed species, except lentils [30,31].

The prevalence of seed protein allergy is not always well documented, often because of the lack of data. Gupta et al. estimated that the prevalence of allergies caused by soybean and wheat in the USA is around 0.4% [30]. The reported prevalence of sesame allergy is around 0.1–0.2% [32], but Osborne et al. estimated a 0.8% prevalence in an Australian pediatric study conducted with 1-year-old infants [33]. Data for lupine and mustard derive from a small number of patients and need to be consolidated.

Other species of seeds such as peas [34] or buckwheat can also be allergenic to varying degrees and are responsible for severe allergic reactions reported in France [35]. Buckwheat was also reported as the causative allergen for 0.8% of Korean patients, appearing as frequently as allergies to wheat or lobster proteins [36]. Some rare cases of food allergy to sunflower seeds [37], poppy seeds [38,39], pumpkin seeds [40], and flaxseed [41] were also reported. An increase in this prevalence can be expected due to recent trends in nutritional behavior, with increased numbers of vegetarian or flexitarian consumers that use these seeds and their proteins as ingredients in their meal composition. Although data on the allergenicity of plant proteins are still sparse, they do suggest that these proteins are important triggers of food allergy reactions all around the world.

## 3. Food Processing and Protein Allergenicity

The allergenic potential of a protein is its capability to sensitize the immune system and trigger an IgE-type reaction [42]. This reaction is triggered at the molecular level by immunoreactive oligopeptide sequences formed by 8 to 12 amino acids (epitopes). They are of two types: (i) linear epitopes, corresponding to continuous amino acids in the protein sequence, (ii) conformational epitopes, formed by discontinuous amino acids in the three-dimensional structure of the protein. Experimental epitope identification is an expensive procedure and comprises several challenges even if helpful computational approaches are employed [43]. Methods using overlapping synthetic peptides, recombinant allergenic fragments, or unfolded allergens to identify linear epitopes permitted the identification of many linear epitopes as reported in IEDB (http://www.iedb.org, 31 May 2020) [44,45,46]. They have been experimentally identified only for few seed allergens, for example, glycinin (Gly m 6) [47]. The conformational epitopes are more challenging; they represent less than 1% of gathered epitopes in the database. Some were reported for peanut (Ara h 2 and Ara 6) or soybean (Gly m 6) allergens [45]. It is noteworthy that among a myriad of dietary proteins, only a small number are responsible for the majority of allergies [31]. The comparison of structure and physicochemical properties of allergens revealed some predictive data related to their allergenic potential. However, for each of them, counter-examples were reported, so that general rules are not applicable [48].

Generally, food processing is designed for improvement of shelf life, functional properties, quality (flavor, texture, taste, and color), preservation, and detoxification. Also, processing can be used to deactivate IgE-binding epitopes and to reduce the adverse effects of allergens in food products [16], so that each food processing step is an opportunity to modify the properties of allergenic proteins. Scientists and the food industry are pushed to develop new technologies and recommend new recipes to reduce the allergenicity of foods and then protect sensitive patients. The strategies to reduce allergenicity are often related to the stability of food allergens to thermal, nonthermal treatments, or proteolysis, as reported for plant proteins in Table 2.

Heat treatments have shown to be able to reduce the allergenicity of thermolabile proteins. These proteins are easily denatured, but these treatments do not affect all allergenic proteins. Peanut allergens are good examples as they may undergo partial to total denaturation, depending on the type of thermal process (roasting, boiling, or frying) and temperature schedules, which may lead to aggregation, and changes in their susceptibility to digestive enzymes and bioaccessibility. Depending on the ingredient and the chemical environment, allergenic proteins may also react with other compounds in the food, by oxidation, disulfide bridge exchange, and Maillard reaction [49,50]. All these changes are likely to affect the epitopes on these allergens and therefore modulate their allergenicity [51]. In the case of plant allergens, studies have shown that heating can reduce allergenicity, but rarely abolish it completely.

In some cases, thermal treatments can be ineffective or even worsen the allergenicity [65,66]. The usefulness of nonthermal processes, mostly used for food preservation and less often for food preparation, in reduction of allergenicity has been less studied. Still, there is an indication that their impact is also highly dependent on the allergen, the food considered, and the settings applied [67,68]. The instant controlled pressure drop (ICPD) process that combines dry steam and heat pressure failed to decrease the IgE immunoreactivity of allergens in lupine and chickpea [59,63].

Another technological option is enzymatic hydrolysis that breaks down the peptide bonds, impacting those that structure the epitopes. This procedure can reduce allergenicity significantly, mainly when carried out extensively [69]. This procedure has been used mostly for the preparation of highly hydrolyzed casein or whey proteins, tolerated by the majority of allergy-sensitive babies [69]. However, some atopic dermatitis-type reactions may persist after the consumption of these hydrolyzates [70]. Concerning plant proteins, several studies have shown that enzymatic hydrolysis can reduce the in vitro IgE binding of the treated allergens. For example, treatment of whole roasted peanuts and peanut flour with proteases has diminished the IgE-binding capacity [71]. On the other hand, hydrolysis of cashew proteins with pepsin resulted in lower IgE reactivity. Hydrolyzed proteins have been used in cashew allergy immunotherapy with success, suggesting that most allergens have been destroyed [72]. Extensive enzymatic hydrolysis is currently the most effective process for reducing food allergenicity. However the application of these enzymes, regardless of type, on a mass scale may be limited by the production costs and also by the negative impact on the functional and sensorial properties of the hydrolyzed ingredient.

## 4. Fermented Foods and Allergenicity

Besides the application for food preservation, fermentation is also used for the transformation of substrates of animal or plant origin into products with new sensorial, nutritional, or functional properties. After cooking, fermentation is the oldest food processing method, and fermented products can be traced back to the Neolithic period between 7000 and 6600 BC [73]. This practice has subsequently spread to the Mediterranean region, for preparation of beverages, such as wine, beer, and other fermented drinks [74,75]. Native American tribes have used fermentation for the preparation/transformation of plant products in the context of their spiritual practices [76].

Fermented food products account for about one-third of the food consumed in the world. The list of fermented products is broad, diverse, and heterogeneous, due to traditions and cultures of the regions of the world they come from [77]. Fermented products from wheat, soy, meat, and a myriad of vegetables, typical in certain parts of the globe, are gaining space in new markets.

Foods are fermented by a variety of microorganisms naturally present or added to the substrate as starter cultures, such as yeasts and bacterial strains, for improvement of taste, structure, nutritional value, and shelf-life [77]. Additionally, fermented food products are good sources of bioactive peptides [78,79,80,81], phenolic compounds [82], and compounds with a rich antioxidant activity [83].

**Figure 2:**
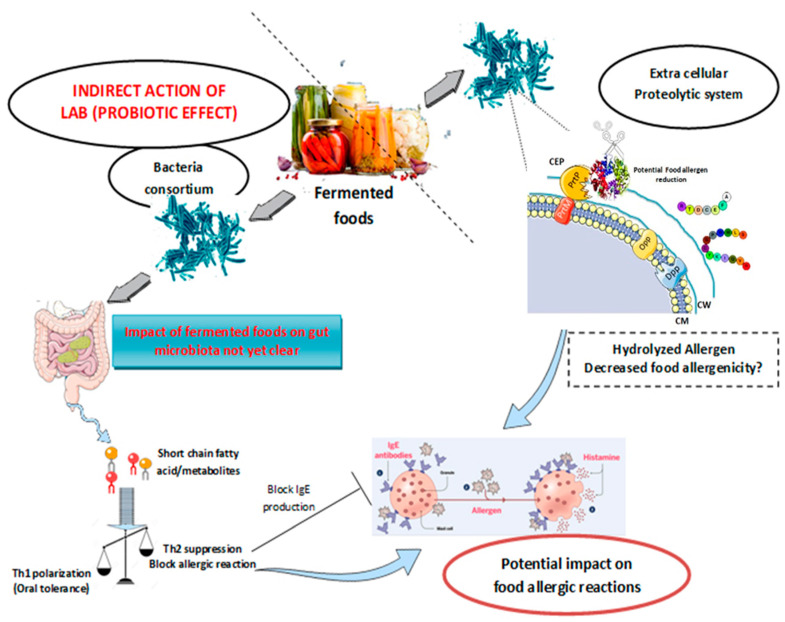
Potential direct and indirect effects of food fermentation by lactic acid bacteria (LAB) on food allergy modulation. The direct effects are represented by the degradation of the allergen by the LAB proteolytic system. The indirect effects are related to the probiotics properties of the fermented foods that could modulate the intestinal microbiota and then prevent food allergy. CW: cell wall; CEP: cell envelope protein; Dpp: dipeptide permease; Opp: oligopeptide permease; PrtP and PrtM: proteinase production promoters.

Lactic acid bacteria (LAB) play an essential role in food fermentation processes. LAB are auxotrophic and depend on their proteolytic system to meet their nutritional requirements for amino acids in food matrices deficient in nitrogen sources necessary for growth. The main components of their proteolytic systems are proteases associated with the bacterial envelope, an amino acid and peptide transport system, and a range of intracellular peptidases [84]. The presence of such complex proteolytic systems allows them to improve nutritional quality (digestibility) of dairy products, such as yogurts, fermented milks, and cheeses, and to shape their sensory properties. The proteolytic activity of LAB can affect allergenic epitopes, reducing the allergenicity of many food components. On the other hand, the fermented food microflora could act as probiotics and modulate the immune system by the direct or indirect impact on the human microbiota. In addition, the proteolytic activity can influence the allergenicity of certain proteins, but the underlying mechanisms were not explored yet (Figure 2).

Proteolytic enzymes have been extensively studied for their technological applications in the development of fermented dairy and meat products. In the last decade, research interest has moved to unveiling the beneficial consequences of the application of proteolytic starter cultures for activity on allergenic proteins and production of specific bioactive proteins. A pivotal point to better understand the proteolytic processes in fermented food products is the identification of the resulting peptides. Peptide transformations were well studied in the dairy products area, highlighting the importance of the functional proteolytic system in different types of cheeses [85,86,87,88]. As a result of the proteolytic activity, some allergenic peptides were biotransformed into inert fragments, while others were transformed into bioactive peptides [89,90]. Starter cultures from the collection of LB Bulgaricum PLC, in Sofia, Bulgaria, and LAB strains isolated from goat milk, were evaluated for fermentation of caseins, α-lactalbumin, and β-lactoglobulin, and results demonstrated the potential application in the production of dairy products from caprine milk [89]. Proteolysis during ripening of Emmental cheese resulted in reduction of allergenic peptides and release of bioactive peptides [91,92,93]. Peptides derived from β-casein, α-casein, and α-lactalbumin have shown to have immunostimulatory activity, which can influence the phagocytic activity [94] and modulate lymphocyte functions [95].

### 4.1. Fermented Dairy Products and Milk Allergy

To better contextualize the importance of seed plant proteins as allergens, some advanced and relevant studies on the allergenicity of fermented dairy products were included in this review. The prevalence of cow milk allergy (CMA) in children remains the highest one, with self-reported rates varying from 1.9% to 2% in the USA, Europe, and China [96,97,98]. When challenge-verified prevalence in the European population is considered, the prevalence percentages decrease to 0.5% to 0.6% [97,99].

Several types of milk-based fermented products have been characterized for their allergenicity. A variety of LAB strains isolated from traditional fermented milk products such as Egyptian cheeses and Iranian camel milk can hydrolyze to varying degrees the major allergenic proteins in milk (caseins mainly) and therefore reduce their antigenicity. Some studies have gone a step further, showing an in vitro reduction of the human IgE recognition toward the considered allergens (Table 3) [100,101,102]. 

A recent study conducted by Wróblewska et al. has shown that the fermentation of sweet buttermilk by *Lactobacillus casei* LcY, an LAB species widely used in agroindustry, was able to eliminate the IgE immunoreactivity towards the four main milk allergens: α-lactalbumin, β-lactoglobulin, β-casein, and k-casein. The authors observed that α-casein remained detectable by the sera of patients after the fermentation process, indicating limited hydrolysis of this protein. This IgE response still decreased after simulated gastric digestion. The authors also found that fermented buttermilk contained two enzymes present naturally in the cell wall/membrane protein fraction of the *Lb. casei* cell that were able to bind to human IgE from milk allergic patients [103]. 

These in vitro studies demonstrate that antigenicity of fermented dairy products can be directly affected by the effective proteolysis of the main allergens. However, this proteolytic activity depends on the hydrolytic susceptibility of the allergens, which correlates with their structural properties. For example, β-lactoglobulin and α-lactalbumin, which are compact and globular proteins, are more resistant to gastrointestinal digestion than caseins [48]. 

The in vivo application of dairy fermented products may be a risk of allergic reactions in young allergic children taking into consideration the type of hypersensitivity, as allergic reactions can be mediated or not by IgE. Uncuoglu et al. studied the effect of extensively heated and fermented bovine milk (yogurt), administered to children below two years of age, on IgE-mediated and non-IgE-mediated CMA [104]. The study included children with IgE and non-IgE milk allergy who reacted to unheated milk after oral challenge and subjected them to the consumption of fermented and baked milk diet during 15 days. The allergic reactions after milk consumption were evaluated by skin prick and atopy patch tests. The authors observed that 15 out of 16 subjects (93%) with IgE-mediated CMA who reacted to unheated milk also reacted to yogurt, whereas 11 out of 16 subjects (68%) with non-IgE-mediated CMA tolerated fermented milk [104]. Similar results were obtained by Monaco et al., who observed that 36% of children with IgE-mediated milk allergy failed to oral food challenge with yogurt and this was associated with positive casein prick test [105]. 

In the light of these observations, it is essential to note that even if in vitro IgE immunoreactivity of fermented dairy products decreases, the clinically relevant effect and the conclusions concerning the hypoallergenicity of fermented products should be explored carefully. Consequently, additional preclinical and clinical studies are still required to assess the real allergic risk and anti-allergic effect of fermented dairy products in link with the allergic clinical phenotype and with the sensitization pattern of allergic patients.

### 4.2. Seeds Protein-Based Products

#### 4.2.1. Soy Fermented Products

Soybean is a legume from the oilseed family that is widely consumed by humans and animals because of its high content in proteins, carbohydrates, and fiber. Glycinin (Gly m 6) and β-conglycinin (Gly m 5) are the two major globulins in soybean, accounting for about 70–80% of the total seed globulin fraction [108]. Soybean is one of the eight most significant sources of food allergens in the USA and Europe [109]. 

Some recent studies have shown that fermentation of soymilk by *Lactobacillus helveticus* and *Enterococcus feacalis* strains reduced the IgE immunoreactivity of patients allergic to the two main soy allergens: Gly m 5 and Gly m 6 [110,111]. A study conducted by Yang et al. indicated that solid-state fermentation of a soybean meal by a starter culture containing *L. casei*, yeast, and *Bacillus subtilis* has impacted the allergenicity when studied by in vitro and in vivo methods. Soy proteins were degraded into low-molecular-weight polypeptides, in which allergenic epitopic sequences of β-conglycinin and glycinin were destroyed, and a decrease in the in vitro human IgE-binding capacity of the fermented soybean proteins was observed [112]. Sensitization experiments in BALB/c mice model revealed that the fermented soybean products induced lower levels of mMCP-1 and specific IgE and a higher level of IFN-γ associated to a smaller degree with the intestine damage as compared with the nonfermented soybean group [112]. 

Soy sauce is a fermented product widely used as a traditional Japanese and Chinese seasoning (shoyu). This product is obtained from soybean and gluten proteins by two-step fermentation, using *Aspergillus oryzae* and LAB strains. The manufacturing process is complex: after fermentation, the product undergoes several steps of heating and filtration, which contribute to the reduction of allergenic potential. However, soybean allergens may not be entirely degraded even after six months of fermentation, as occurs in Moromi, a type of soy sauce submitted to long fermentation. Immunoblotting using an anti-soybean IgG antibody and the sera from two children with soybean allergy indicated that the soy allergens remained in this product after the fermentation. However, the allergens were undetected after pasteurization and ultrafiltration, probably due to the thermal denaturation and retention after ultrafiltration step. The authors of the study also assessed the presence of some residual soy allergens in different commercial soy sauces, immunostained by patient sera, further identified as the β-subunit of β-conglycinin and oleosin, which are not known as major soybean allergens [113]. 

#### 4.2.2. Other Cereal-Based Fermented Foods

*Triticum aestivum* (bread wheat) is the most widely grown crop worldwide. In some predisposed individuals, wheat can cause inflammatory immune reactions represented by celiac diseases and wheat allergy or intolerance known as non-celiac gluten/wheat sensitivity with currently unclear mechanisms [114]. The prevalence of wheat allergy is about 0.1% [24], caused by IgE or non-IgE hypersensitivity reactions, triggered by different wheat proteins. The enzymatic proteolysis is not very effective for reduction of the allergenicity of wheat proteins because this process damages the functional properties of gluten proteins, necessary for bread-making, so that fermentation with proteolytic LAB strains is an alternative to reduce the allergenicity of these proteins.

Stefańska et al. identified eleven strains of LAB isolated from leaven capable of hydrolyzing gluten proteins and albumins/globulins. In vitro tests indicated that immunoreactivity against human IgE was reduced, but a residual allergenicity remained, even after simulated gastric digestion. The authors concluded that the proteolytic activity of the selected LAB strains was insufficient for use in wheat flour bakery products intended for patients with allergy to gluten [115]. Rizello et al. also addressed this issue, evaluating the impact of wheat and rye flour fermented by LAB on the allergenicity of sourdough bread, observing a significant reduction in the reactivity of IgE from wheat-allergic patients. Regrettably, the effect of yeasts in the fermented products and the contribution of fungal proteases in the proteolytic activity were not discussed [116]. Despite the use of different experimental conditions, these two studies indicated that the immunoreactivity of fermented products toward the human IgE from wheat-allergic patients was reduced, but their residual allergenicity needs still to be confirmed by ex vivo and/or in vivo tests.

Wheat allergens may also be present in soy sauces as added ingredients, posing a potential risk of allergic reactions. The impact of fermentation during soy sauce maturation on wheat allergens’ degradation was evaluated by Kobayashi et al., who used in vitro methods using sera from five children with a wheat allergy. These authors observed that allergens were degraded and lost their IgE-binding ability in both salt-soluble and salt-insoluble fractions of soy sauce during pressing or fermentation stage [117]. Considering the diversity of soy sauce processing, more studies are required to conclude on the safety of soy sauce for wheat-allergic patients.

### 4.3. Modulation of Immune Responses by Fermented Foods: In Vivo Studies

Most studies that investigated the impact of fermented foods on allergies were performed using in vitro models, and results rarely derive from preclinical or clinical studies, except for some observations of probiotic effects. The details of the mechanisms by which these foods or their components modulate the physiological functions or the homeostasis of the organs related to these health benefits are often unknown.

Wróblewska et al. tried to assess the probiotic properties of whey proteins fermented with *Streptococcus salivarius* subsp. *thermophilus* 2K and *Lactobacillus delbrueckii* subsp. *bulgaricus* BK–FW. The study was carried out using a mice model with induced cow milk allergy. Interventional feeding of α-caseins- and β-lactogloblulin-immunized mice with fermented whey changed Th_1_/Th_2_ balance towards a Th1 response and enhanced the secretion of regulatory cytokines, and reduced allergy markers’ level [118].

In a Korean study [119], the link between the consumption of local fermented foods (kimchi, doenjang, chungkookjang, fermented seafood) and the prevalence of atopic dermatitis (AD) was evaluated. In a cohort of healthy Korean patients with atopic dermatitis (9763 adults), participants were divided in four groups according to their diet and the daily frequency of consumption of different types of foods: processed foods, fruits and vegetables, fermented foods, and meat. The authors observed a positive correlation between consumption of meat and processed plant products and the appearance of AD, and a negative correlation when fermented foods were consumed more than 92 times per month (Odds Ratio, 0.56 % confidence interval (CI), 0.37–0.84), with less AD found. The results indicate a potential prophylactic effect of fermented products against atopic dermatitis in Korean population.

Another Korean study carried out by Lim et al. on the animal model of atopic dermatitis demonstrated that kimchi (fermented mustard leaf) decreased the symptoms related to AD compared with controls. At the molecular level, *Weissella cibaria* WIKIM28, the dominant bacterium in kimchi, has been shown to be able to direct the immune response towards an anti-inflammatory and tolerogenic reaction [120].

On the other hand, Hong and Chen demonstrated in a preclinical study that oral feeding of heat-inactivated *Lactobacillus* (Lb.) *kefiranofaciens* M1 isolated from kefir grains had inhibited effectively immunoglobulin (Ig) E production in response to ovalbumin (OVA) challenge in vivo using an animal model. Also, the pattern of cytokine production by spleenocyte cells revealed that the levels of cytokines produced by T helper (Th1) cells increased and those of cytokines produced by Th_2_ cells decreased in the heat-inactivated M1 feeding group. These findings indicated that *Lactobacillus kefiranofaciens* M1 played an important role in anti-allergic activities [121].

These studies indicate that the microflora in Asian fermented foods play an important role as therapeutics or preventives against developing food allergies or their symptoms.

## 5. Conclusions

Besides the nutritional value, fermented food products provide additional health benefits. Yet, the scientific arguments and evidences are often scarce, and more intervention studies using fermented foods and probiotics to prevent or treat allergy/atopic diseases are needed.

Apart from bakery products, the consumption of fermented products based on plant proteins is relatively limited in Western countries. The increasing interest in the use of vegetable proteins, fermented or not, as alternatives to animal proteins imposes stricter control of their allergenicity and especially their claimed hypoallergenicity.

Based on the several studies reviewed here, it is clear that more data are needed for science-based conclusions of the real impact of fermentation on the allergenicity of foods. The knowledge of the molecular structures of allergens and their fate during fermentation is essential for understanding the fermentation impact. New methods such as the use of recombinant allergens or synthetic peptides could be helpful to go further. Given the complex composition of fermented foods, it is essential to investigate how each nutrient and/or metabolite derived from the fermentation process contributes to the management of food allergy risks. The complexity of food composition raises several questions that remain unanswered: (i) Do probiotic strains prevent food allergy by consolidation of the intestinal barrier permeability or by influence in the immune system only? (ii) Is supplementation of probiotic strains with proteolytic activity sufficient to abolish the allergic reactions in vivo? (iii) Does the presence of partially hydrolyzed allergens favor tolerance development? (iv) What are the impacts of a complex fermented product, including a predigested matrix, metabolites, and microorganisms, on allergic reactions and can these elements act in synergy?

These different points should be studied intensely. Standard tools such as IgE-binding capacity measurement, ex vivo studies (cellular models mimicking some phases of the allergic reaction), and also in vivo studies (particularly with animal models) should be performed. Additionally, clinical studies should be carried out to validate the use of fermented foods as hypoallergenic products for allergic patients or to evaluate their capacity to modulate the immune system by inducing tolerance in atopic patients.

Once the health benefits regarding allergy are validated, fermented foods could be included in the nutrition guidelines for the population, particularly for vulnerable consumers with chronic diseases, such as diabetes, allergy, celiac diseases, and obesity.

## Figures and Tables

**Table 1:** List of seed allergens, according to WHO/IUIS allergen nomenclature (http://www.allergen.org/search.php) and Allergome database (http://www.allergome.org/script/search_step1.php?clear=1).

	Cupin		Prolamin				Profilin	PR
	11S Globulin	7S, Vicilin, Vicilin-Like	2S Albumin	Prolamin	LTP, ns LTP	Amylase/Trypsin Inhibitors		
Wheat (*Triticum aestivum*; *T. turgidum* ssp.; *T. urartu*)				Tri a 19, Tri a 20, Tri a 21, Tri a 26, Tri a 36, Tri tp 26, Tri ur 26	Tri a 14 Tri tu 14	Tri a 15, Tri a 28, Tri a 29, Tri a 30, Tri a 40, Tri tu 28, Tri ur 28, Tri tu 30	Tri a 12	
Soybean (*Glycin max, G. soya*)	Gly m 6 Gly s 6	Gly m 5 Gly s 5	Gly m 8				Gly m 3	Gly m 4
Peanut (*Arachis hypogea, A. ipaensis*)	Ara h 3	Ara h 1	Ara h 2 Ara h 6 Ara h 7		Ara h 9 Ara h 16 Ara h 17 Ara h 9		Ara h 5	Ara h 8
Sesame (*Sesamum indicum*)	Ses i 6 Ses i 7	Ses i 3	Ses i 1 Ses i 2				Ses i 8	
Brazil Nut (*Bertholletia excelsa*)	Ber e 2		Ber e 1					
Hazelnut (*Corylus avellana*)	Cor a 9	Cor a 11	Cor a 14		Cor a 8		Cor a 2	
Lupine (*Lupinus angistifolius*, *L. albus; L. luteus*)	*Lup an alpha*	Lup a 1	*Lup an delta ^(1)^*				Lup a 5	Lup a 4 Lup l 4
Buckwheat *Fagopyrum esculentum*		Fag e 3 Fag e 5	Fag e 2					
Mustard (*Sinapis alba, Brassica juncea, B. campetris, B. rapa*)	Sin a 2		Sin a 1 Bra j 1 Bra r 1		Sin a 3 Bra r 3		Sin a 4 Bra j 8	
Rapeseed (*Brassica napus*)			Bra n 1				*Bra n 8*	
Pea (*Pisum Sativum*)		Pis s 1 Pis s 2			Pis s 3		Pis s 5	Pis s 6
Sunflower (*Helianthus annuus*)			*Hel a 2S*		Hel a 3		Hel a 2	
Pumpkin (*Cucurbita maxima*)	Cuc ma 4		Cuc ma 5				*Cuc ma 2*	
Poppy seed (*Papaver somniferum*)							*Pap s 2 *	*Pap s 1*
Lentil (*Lens Culinaris*)		Len c 1			Len c 3			

*Underlined Italic codes*: allergens registered only in the allergome database. PR: Pathogenesis-related proteins.

**Table 2:** Impact of food processing on some plant food allergenicity as assessed by IgE reactivity.

	Process	Heat Treatment				Hydrolysis		Other Treatment
Allergen		Boiling	Roasting	Microwave	Autoclave	Enzymatic	Fermentation	Instant Controlled Pressure Drop
Peanut (*Arachis hypogaea*)		↓IgE reactivity [52]	↑IgE reactivity [53]		↓IgE reactivity [54]	↓IgE reactivity after roasting/boiling No results on raw kernel [55]		↓IgE reactivity at extreme conditions [56]
Soybean (*Glycine max*)		↓IgE reactivity [57]				↓IgE reactivity: (β Conglycinin) ≈ No effect: (glycinin)	↓IgE reactivity [58]	↓IgE reactivity [59]
Wheat (*Triticum* sp.)		≈ No effect [60]					↓IgE reactivity [61]	
Lupine (*Lupinus angistifolius*)				≈ No effect [62]	↓IgE reactivity [62]			↓IgE reactivity at extreme conditions [63]
(*Lupinus albus*)				≈ No effect [62]				
Lentil↑ (*Lens culinaris*)		≈ No effect [64]			↓IgE↓ reactivity [64]			Moderate reduction of IgE immunoreactivity [59]

↓: indicates increase in the IgE reactivity toward the treated allergen; ↑: indicates decrease in the IgE reactivity toward the treated allergen. IgE: type E immunoglobulins.

**Table 3:** Main studies on the effect of fermentation of dairy products and its impact on their antigenicity and allergenicity.

Food/Allergen	Strain	Conditions	In Vitro Analysis	In Vivo Tests	Reference
Na-caseinate and αS1-casein	*Enterococcus faecium*	Non proliferative system	Decrease of IgE binding		[102]
Sweet buttermilk	*L. casei LcY*	Fermented system	Decrease of IgE binding towards all milk allergens. Reactivity to new proteins isolated from cell wall membrane.		[103]
β-lactoglobulin	*Lactococcus lactis*	Non proliferative system	Decrease of IgE binding		[106]
β-lactoglobulin	*Lactobacillus delbrueckii subsp.bulgaricus CRL 656*	Non proliferative system	Decrease of IgE binding		[100]
Milk	*Lactobacillus helveticus*	Fermented system	Unmodified IgE binding toward β—lactoglobulin		[107]
Fermented milk	N.S.		Decrease PT reactivity, decrease IgE β—lactoglobulin and lactalbumin.	Yogurt tolerated by subjects tolerating heated milk	[104]
Fermented milk (Yogurt)	N.S.			Succeed of oral food challenge with fermented food.	[105]

N.S.: not specified. PT: Prick Test.
